# Automatic reconstruction of a bacterial regulatory network using Natural Language Processing

**DOI:** 10.1186/1471-2105-8-293

**Published:** 2007-08-07

**Authors:** Carlos Rodríguez-Penagos, Heladia Salgado, Irma Martínez-Flores, Julio Collado-Vides

**Affiliations:** 1 Programa de Genómica Computacional, Centro de Ciencias Genómicas, Universidad Nacional Autónoma de México, Apdo. Postal 565-A, Avenida Universidad, Cuernavaca, Morelos, 62100, Mexico

## Abstract

**Background:**

Manual curation of biological databases, an expensive and labor-intensive process, is essential for high quality integrated data. In this paper we report the implementation of a state-of-the-art Natural Language Processing system that creates computer-readable networks of regulatory interactions directly from different collections of abstracts and full-text papers. Our major aim is to understand how automatic annotation using Text-Mining techniques can complement manual curation of biological databases. We implemented a rule-based system to generate networks from different sets of documents dealing with regulation in *Escherichia coli *K-12.

**Results:**

Performance evaluation is based on the most comprehensive transcriptional regulation database for any organism, the manually-curated RegulonDB, 45% of which we were able to recreate automatically. From our automated analysis we were also able to find some new interactions from papers not already curated, or that were missed in the manual filtering and review of the literature. We also put forward a novel Regulatory Interaction Markup Language better suited than SBML for simultaneously representing data of interest for biologists and text miners.

**Conclusion:**

Manual curation of the output of automatic processing of text is a good way to complement a more detailed review of the literature, either for validating the results of what has been already annotated, or for discovering facts and information that might have been overlooked at the triage or curation stages.

## Background

Genomics and Systems Biology rely on vast amounts of organized data in order to use the sophisticated bioinformatics tools that allow modeling, analysis and interpretation of biological processes like gene regulation, variation in expression profiles and metabolic pathways [1]. Gene regulation, for example, involves complex interactions between genes, transcriptions factors (TFs), proteins and metabolites that can be visualized as networks of inducing and repressing interactions controlling gene expression. Those interactions triggering gene expression and protein synthesis may control cell development and adaptability to environmental changes. Most of the end result of this kind of biological research materializes in textual publication in peer-reviewed journals, but as such is not directly amenable to computational treatment. Databases compiling this information have been developed for some organisms (for example EcoCyc [2] for *E. coli*, or SDG [3] for *S. cerevisiae*). However, the manual curation involved in ensuring the reliability of this data is a resource- and labor-intensive endeavor. The ever-expanding literature (represented by the millions of papers in electronic repositories that have come to be known as the *bibliome *[4]) can literally overwhelm the ability of researchers to make sense of this flood of information. Thousands of papers must be selected and retrieved from repositories such as NIH's PubMed and Medline, and carefully reviewed by experts in order to extract the facts needed by the research community. The last ten years have seen a proliferation of Natural Language Processing (NLP) techniques to aid in dealing with the explosive growth of useful data. Several good overviews [5-8] of the state-of-the-art in Text-Mining have been published recently, and some of the techniques have reached an acceptable level of maturity that allows them to finally perform reasonably well. Information Extraction (IE) is one of the computational methods that has been used successfully before in other non-scientific fields [9]. In contrast to Information Retrieval, which provides a ranked list of relevant documents, IE not only finds which sources of information are relevant, but also automatically populates databases with information that fulfills the user's needs. Compilation of the extremely complex networks of biochemical interactions that control developmental and functional processes in cells is a prime example of the usefulness of these technologies for biological research, as evidenced, for instance, in the various systems competing in the BioCreative evaluations [10], or in earlier examples, the PastaWeb [11], GENIES [12], iHOP [13] and BioRat [14] systems, all of them correlating biological networks to the literature that describe them.

In this work, we show how, and to what extent, a state-of-the-art NLP system can aid the manual curation of transcriptional regulation in *Escherichia coli*, and how both approaches (manual vs. automatic annotation) can complement each other and enrich curation. Although we don't claim any significant technical advance in the current state-of-the-art in text mining *per se*, we present a much-needed evaluation of the impact of Human Language Technologies (HLT) on annotation and data-gathering using as a gold standard a biological model (a regulatory network) instead of an annotated corpus. We also suggest a methodology to merge these methodologies into the conventional curatorial workflow.

## Results

For the overall architecture of the IE system, we adapted a rule-based pipeline first described by Saric *et al*. for the STRING-IE system [15,16]. The pipeline is described in detail in the Methods section. We chose a Language-Engineering approach since in general we were concerned more with accuracy (precision) than with coverage (recall), and wanted to be fairly sure about the regulatory interactions we would be extracting. Rule-based systems are more accurate for well-defined tasks (although they are very labor-intensive to implement), while statistically-based approaches, although less exact, are more robust and tolerant to noisy data and errors.

In order to test the capabilities of the extraction system reported here, various collections of documents were downloaded; involving both abstracts from PubMed or full text articles available from the various journal's websites or from subscription services. We employed different strategies for the triage process, which will be described in the Corpus subsection. In this work we used fairly standard search procedures, either gathering the lists of references from pre-curated databases, or doing various keyword searches using the NIH *Entrez *facilities.

**Figure 1:**
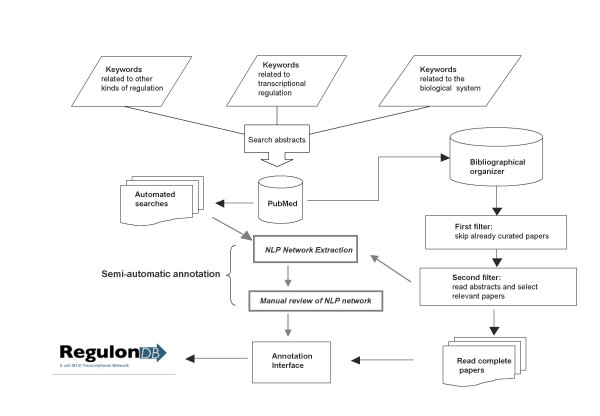
**Annotation workflow**. A suggested workflow for parallel manual and automatic annotations of transcriptional regulation, with manual review of automatically-generated networks in shaded lines. Curators would check the interactions mined from text, since they would be provided with the reference papers and the textual segments from which the system retrieved them.

The database we used to evaluate the output of our IE system, RegulonDB, [17] is the primary source of curation of original literature with experimental knowledge about the elements and interactions of the network of transcriptional regulation in *E. coli *K-12. RegulonDB can be considered a computational model of mechanisms of transcriptional regulation in this organism, and contributes data to the EcoCyc database. Both contain mechanistic information about operon organization and their decomposition in transcription units, promoters and their sigma type, binding sites of specific transcriptional regulators, etc. The usual curation process starts by searching for articles that contain information about transcriptional regulation (Figure 1), using a set of pertinent keywords in the PubMed database. To select only the most relevant articles, a team of biologists-curators reads the abstracts of these papers. Other papers not originally retrieved with our search strategies are suggested by external researchers and are incorporated into the curation cycle. Finally, the data annotated by reading the articles is added to both RegulonDB and EcoCyc databases. Although this curation process yields reliable data reflecting what is there in the literature, it is a long-term and manpower-intensive effort in which errors can occur at different stages, and recovery of omitted data is very difficult. Furthermore, with high throughput technologies, the amount of published interactions with some type of experimental evidence (a requisite in our database curation process), will likely scale up in the near future. This motivates us to get prepared for new curation strategies combining automatic NLP processes with human curation.

### Corpora

**Figure 2:**
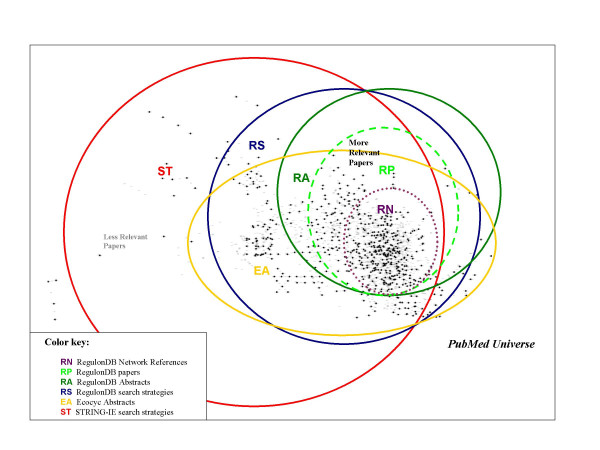
**Corpus coverage of transcriptional regulation in *E. Coli***. Venn diagram illustrates overlapping coverage in corpora used in this work, with dots representing papers relevant for transcriptional regulation in *E. coli *K-12. Different selection strategies (keyword searches on PubMed and curated databases references) result in diverse document sets, which can contain in some cases groups of the same documents as well as other non-relevant papers.

For our evaluation of curation of *E. coli *regulatory networks we collected different sets of abstracts and full papers that we knew contained, to varying degrees, valuable information on the subject of transcriptional regulation. Some sets were based on manual curation efforts from related databases (RegulonDB and EcoCyc), while others resulted from carefully-crafted search strategies using the NCBI PubMed facilities, so that those manually-selected ones constitute a reliable baseline of positive examples of information-rich documents. The different corpora used in our study are summarized in Table 1, ranked (in column 1) according to their putative relevance to the domain of transcriptional regulation in *E. coli *K-12. The corpora include both full-text papers and abstracts, and range from around 13,000 abstract document sets from RegulonDB curator's keyword searches (RS) or the EcoCyc [2] database (EA, more focused on metabolic pathways and genomics than RegulonDB) to 724 full-text papers (RN) carefully selected by RegulonDB curators as containing information on the regulatory network. One of the corpora (ST) was compiled by Saric *et al*. [16] by searching the full PubMed for abstracts that mentioned *E. coli *and sentences that contained at least two gene names, a strategy that although could find many relevant articles but would nonetheless introduce a lot of noise due to the nature of the bacteria as a model organism used in many biological contexts. The different selection criteria of the diverse datasets analyzed reflect different purposes, and a comparison of the results obtained using each one, as a textual source, would be of obvious interest for text miners and curators alike. Figure 2 illustrates in a Venn diagram how each of the six corpora might overlap and complement each other. The dots represent papers putatively relevant for transcriptional regulation in our bacteria, and the different selection criteria (keyword searches on PubMed and curated databases references) result in diverse document sets, which can contain groups of the same documents as well as other papers also relevant. Thus, corpus RN with curator-reviewed references to the regulatory network constitutes the most relevant textual source, while corpora ST and EA contain less pertinent, more diverse information. A search with a wider net cast, for example, in the ST corpus will contain many more papers than the EA one, or the RS one (which uses the keyword search algorithm developed for curating transcriptional regulation), but the quality and density of relevant information will supposedly be higher in the human-selected RA or RP corpus. Full-text papers are logically richer in information than abstracts, since the latter constitute only brief outlines of the main claims of papers and lack the concrete detailed data that mining efforts strive to extract from documents. One of the purposes of using such a diverse array of corpora was to see if the added effort needed to use full-text papers for text mining was worth it, as well as getting some insight on which search strategy to locate relevant documents was more valuable.

**Table 1:** Document sets (corpora) used in this work

	**ID**	**Name**	**# of docs**	**size in MB**	**type**	**description**
**1**	**RN**	RegulonDB Network References	724	24.9	full-text	Full-text papers from the RegulonDB database references that curators have identified as referring specifically to the regulatory network, as opposed to those referring to other objects from the database.
**2**	**RP**	RegulonDB papers	2,475	99	full-text	Full text papers from the complete RegulonDB references that we were able to access and download.
**3**	**RA**	RegulonDB Abstracts	3,075	3.3	abstracts	Abstracts from the complete RegulonDB references, as of June of 2006.
**4**	**RS**	RegulonDB search strategies	12,059	12.3	abstracts	Corpus generated by using the RegulonDB curator's search strategies, without any subsequent filtering.
**5**	**EA**	EcoCyc Abstracts	13,334	14.4	abstracts	Abstracts from references in the 2006 EcoCyc database that describes the genome and the biochemical machinery of *E. coli*.
**6**	**ST**	STRING-IE	58,312	10.7	sentences	Corpus of distinct sentences generated by the STRING-IE team by searching in PubMed for "*E. coli*" (and synonyms), and two gene/protein names in the same abstract, from 195,000 abstracts.

Description of the different full text and abstract corpora used for extraction of regulatory interactions. The document sets are based on PubMed searches and on reference lists from database curation efforts.

### A markup language for mining bacterial regulatory networks

The system's output is an xml file with a format we have called *Regulatory Network Mining Markup Language *(or RNM^2^L), which allowed representation, in a user-friendly way, of the basic data relevant to Information Extraction of genetic regulation, both from the perspective of a biologist's interests (what genes were activated/repressed by which transcription factor/protein) and from the NLP specialist's needs (where was this information retrieved from?, what linguistic rule was triggered in its retrieval?, etc.). Interactions are here defined as a TF-target gene relationship, independently of the number of binding sites of a TF upstream of a particular promoter. The following example shows some of the main features of a typical entry in RNM^2^L from our tests, depicting sentence (1) as a source.

(1) In contrast, acnB expression is activated by CRP and repressed by ArcA, FruR and Fis from PacnB.

In the "interaction" node, the label *from *shows the internal name of the final extraction grammar rule that justified retrieval of that sentence, while *source *refers to the collection where it was found, and *pmid *refers to the identification number of the paper where this particular sentence was retrieved from:

<interaction ID="596" from="anaph+ev_act_expr_xr" ri_function="repressor" source="RegulonAbs">

<regulator GenProtID="ECK120011345" org="ecoli" type="nxpg"> **ArcA **</regulator>

<regulated GenProtID="ECK120002193" org="ecoli" type="nxpg"> **acnB **</regulated>

<evidence verb="repressed"/>

<sentence pmid="9421904"> In contrast, acnB expression is activated by CRP and repressed by ArcA, FruR and Fis from PacnB. </sentence>

</interaction>

Thus, we identify two entities, one the regulator and the other the regulated one, with unique ID numbers for each, as well as organism and semantic types (here "nnpg" means: *either a gene or a protein*, as sometimes they cannot be disambiguated fully). Note that this entry represents only one of the four different one-to-one interactions that can be extracted from the sentence. We devised RNM^2^L because: A) System Biology Markup Language [18] was considered too cumbersome for the more limited task of representing networks with discrete states, and B) because a more specialized tagging scheme would allow better comparison between networks extracted from different sources or with different systems or methodologies, while at the same time being useful both for NLP optimization and for biological interpretation. In the supplementary material site we provide a defining DTD schema for RNM^2^L.

### Evaluation criteria for the network extraction task

To evaluate the extracted regulatory networks we implemented a benchmarking tool that checked each interaction against the RegulonDB database. We assume the database is a reliable gold standard of the final desired output, in contrast to the more customary evaluation methodologies that use a set of manually-annotated sentences from which a database of relevant information can be elicited. Since our database does not include the actual sentences from which the facts have been extracted, our automatic evaluation does not have available to it the linguistic expression of the facts. This methodology involves, of course, some assumptions about the completeness and exhaustiveness of the RegulonDB database. Since *E. coli *K-12 is one of the best understood model organisms in the literature, and the RegulonDB is carefully compiled and monitored, we can nevertheless rely on it to be a good baseline for evaluation.

Those interactions that had a regulator gene/protein not found in RegulonDB (either by ID, name or synonym) were filtered out, but overall we kept interactions where the gene/protein was identified as a mutant or coming from another (mainly bacterial) organism. Our evaluation considered two cases, a) where the interaction has both the regulator and the regulated genes/proteins correctly identified but their type of interaction might be undetermined, or b) where, besides a correct identification of the involved entities, the nature of their interaction was also correctly identified (as activation or repression).

To test how previously-known information on the network could support the extraction effort, we created two different networks from each corpus: one where we artificially inserted interactions derived from previous knowledge about operons, heterodimers and the like, and another one where we assumed we didn't have *a priori *information about those multi-entity objects; for example, from the sentence: "transcription of the *E. coli melAB *operon is regulated by the MelR protein" the fact that *melA *and *melB *are regulated by the MelR protein could be extracted. The non-enriched networks, although less complete, would be more realistic with regard to the true capabilities of the extraction system, when applied to other less studied organisms for which we don't have as much genomic information as for the *E. coli *proteobacteria.

We calculated precision, recall and F-measure of the extracted samples by considering RegulonDB as the ultimate instance of the bacterial network. We previously subtracted from it all computational predictions, since they would conceivably not be present in experimental papers. Recall (the number of relevant interactions extracted with regard to all possible relevant interactions that should have been retrieved) was estimated using the entries in our reference RegulonDB database, and considering as a single one the cases where there was both activation and repression annotated. This is not an orthodox measure of exhaustiveness in Information Extraction evaluations, but since we were not using annotated corpora it was the closest we would get to knowing how many extractable phrases were extant in our corpus, assuming all were there and available to the system. Because of these assumptions, the actual performance of the system could realistically be significantly better than what this fully-automatic verification might lead to believe. For precision (accuracy, or the ratio of all relevant interactions extracted vs. number of all interactions retrieved), we calculated two values: one (precision 1) where we used all the interactions retrieved (even if they were filtered out because the regulator gene/protein was not found in RegulonDB), and another one (precision 2) in which we only considered the subset of all interactions where the regulator gene/protein was cataloged in the reference database. As in other aspects of these evaluation, we did this in order to understand what would happen in cases where we are dealing with other organisms for which we don't have all the information that we have available for *E. coli*, for instance, where we don't have an assumption of a completely curated network or not all transcription factors are known.

In addition to the networks extracted from the different document collections described earlier in the Corpora section, we also integrated into a single network all unique interactions retrieved from all the text-mining sources (AS, in Table 2), in order to have a single sample that would be as exhaustive as possible, regardless of whether the initial search strategy was fine-grained (as in the manually-selected RegulonDB sets) or coarse-grained as in the 195,000 abstract corpus from STRING-IE keyword searches. Our final metrics for various datasets are shown in Table 2, and the network files, customized processing modules and supplementary material can be found at the RegulonDB website [19].

**Table 2:** Final network extraction system evaluation metrics

**Source**	**(1)**	**(2)**	**(3)**	**(4)**	**(5)**	**(6)**	**(7)**	**(8)**	**(9)**
**RegulonDB **(as of June 2006)	3108	-	3397	3397	100%	100%	-	-	-
**AS**	3148	768	661	1429	45%	45%	0.45	0.77	0.57
AS*	2649	569	535	1104	41%	35.5%	0.41	0.72	0.47
**RP**	2650	711	605	1316	49%	42%	0.49	0.78	0.55
RP*	2202	522	491	1013	46%	32%	0.46	0.74	0.45
**RN**	1643	555	471	1026	62%	33%	0.62	0.85	0.47
RN*	1354	426	385	811	59%	26%	0.59	0.81	0.39
**EA**	627	262	140	402	64%	12%	0.64	0.95	0.22
EA*	554	217	114	331	59%	10%	0.59	0.91	0.19
**RS**	718	254	146	400	55%	12%	0.55	0.91	0.22
RS*	630	207	121	328	52%	10%	0.52	0.86	0.18
**ST**	691	199	143	342	49%	11%	0.49	0.90	0.19
ST*	628	170	118	288	45%	9%	0.45	0.86	0.16
**RA**	414	207	115	322	77%	10%	0.77	1.00	0.18
RA*	370	180	97	277	74%	8%	0.74	0.99	0.16

An asterisk [*] next to source name indicates that no multiple-unit objects (like the individual elements of two-system components, or operons) were added; The RegulonDB "dual" interactions (that is, presenting both activation and repression), are counted here as two distinct interactions. **AS **represents a file containing all compiled interactions found in all textual sources used in this work, and constitutes the sum of all non-redundant interactions extracted from the full-text and abstract documents.

1. Unique, non-repeated interactions found for each file

2. Interactions that match RegulonDB interaction pairs, but whose function (activation or repression) was not determined by the system.

3. Interactions that match RegulonDB entries, and also match repressor/activator function

4. Overall matches (column 2 + column 3), including both interactions with complete information as well as under specified ones

5. % of total interactions in file which are correct: (1)/(4)

6. Recall: percentage of RegulonDB's 3108 interactions that is represented by correct interactions in file: ((4)*100)/3108

7. Precision 1: Number of overall matches (4)/unique interactions in file (1)

8. Precision 2: Number of overall matches (4)/interactions in file that contain a RegulonDB-annotated regulator

9. F-Measure (F = 2RP/R+P)

## Discussion

**Figure 3:**
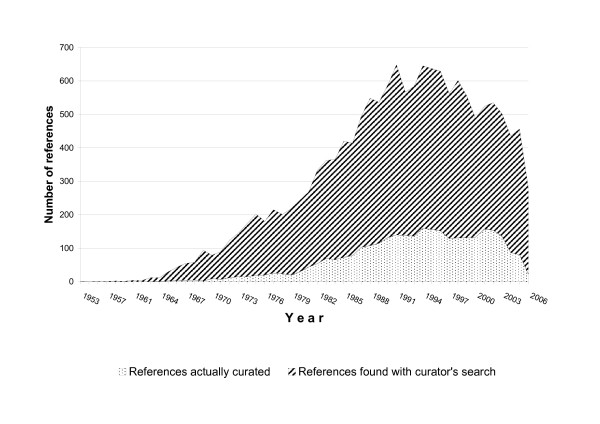
**Curated and retrieved articles for RegulonDB, by year**. Comparison between all references initially retrieved from PubMed using RegulonDB curator's search algorithms, and references that were finally reviewed in full to populate the database. Since search algorithms are refined and changed continuously, this is shown only for illustration.

Our usual curatorial workflow (Figure 1) leaves out between 75 and 50% of all papers retrieved from keyword searches (although this ratio has fluctuated with time). Figure 3 shows how many abstracts were retrieved with our search strategies each year, and how many of them were actually manually curated. One of the motivations of this work was to estimate if our triage and curation efforts were retrieving all the potential information on the subject of transcriptional regulation on *E. coli *that was available in PubMed-based literature, and how to better explore for curation the full extent of the literature obtained in our keyword searches.

A biologist reviewed a random sampling of the most comprehensive set of interactions, which our system was able to extract, but that were not found to be in RegulonDB. This exercise allowed us to explore information that: a) was not retrieved during the manual curation process, but should have been, b) was processed incorrectly or c) although basically correct, was not completely relevant to our current purposes (for example, gene regulation other than transcriptional, although RegulonDB plans to add this information in the future). From a random sample of 96 interactions, we found 19 that represented relevant information that was not present in our reference database, but that merited either a closer look at the sources or further analysis to establish if it should be incorporated into RegulonDB. There were multiple reasons for this information being missing, among them: 1) the source papers had not been retrieved for curation or had not been curated yet, or 2) the genes or TFs were mentioned with unusual synonyms, Ids, references or terms, which made their manual curation difficult, 3) or the evidence presented either was deemed insufficient by curators or was presented with high level of hedging ("the *molR *gene probably regulates the expression of the *chlD *operon"). If we extrapolate this figure to the 1,545 interactions from the comprehensive non-enriched 2,649 interactions set that could not be matched with the relevant 3,108 RegulonDB entries, around 290 new interactions could be added to the database after manual review. RegulonDB curators will curate this automatically-generated network to see if they can integrate the data into the database, but also will search for other pertinent information, such as site and distances for gene and promoters.

In order to test the linguistic processing of the system, we did a manual review of a random sample of 96 interactions extracted, and we established 81 of them as having a basically correct semantic interpretation of the sentences, and 76 of them as being biologically correct to the point of including the right activation or repression function, for a 84% overall precision. The network that was gathered from all sources allowed us to obtain 45% of the entire human-curated RegulonDB network, while a more limited 700-plus selection of network-related papers (RN) accounted for 33% of that total. The "artificial" addition of multiple-entity objects like operons and two-component protein systems from RegulonDB (information that was available from our reference database, but would not be for organisms not yet curated) increased the size of the global network by 10 percentile points (324 interactions). In most datasets the increase was less significant, and we believe that as a whole the value of the information added with previous domain knowledge was not overly important.

In a more extensive evaluation simulating full curation of NLP-generated networks, a domain expert reviewed 481 interactions that A) were obtained from processing the 12,059 abstracts retrieved using the RegulonDB search strategies and B) were not found in the RegulonDB database. Again, we wanted to test if the extraction system could find relevant information that was missed at the triage or curation stages (a difficult task for human curators). We found 91 interactions that could be added to RegulonDB, while in the rest there was either an error in the inference made from the text, or even if the inference was correct the data could not be added to RegulonDB because of various reasons, among them: the regulation was not transcriptional, did not correspond to *E. coli*, there was an error in the gene/protein identification module, etc. In a few of the interactions the data seemed correct but the complete papers were unavailable to check them fully before adding them to the database. The 18.91 % of total interactions that was either immediately useful or seemed correct pending further analysis constitutes a reasonable addition to the curated network that seems to be worth the review effort by the curatorial team (potentially, close to 290 new interactions could be added if we consider the combined network obtained from all our corpora). In any retrieval system, the small "tail" of valuable missed information is always harder to come by than obvious cases, and improvement on Recall and Precision is hard. It is clear that a fully automatic processing of these sentences would require complex Artificial Intelligence inference engines customized for biological interpretation. Data that was not included in the RegulonDB network we tested against (like other regulation mechanisms or plasmid genes) could nonetheless provide important relationships associated with the annotation process. By doing a manual sweep of computer-generated curation, new or relevant information can be garnered that complements, expands or confirms human annotation.

An interesting issue to explore is why our IE system didn't obtain a more complete network from these papers. In other words, what are the limitations of an extraction system such as the one implemented for this task? First, there is the issue of the availability of full-text papers, which contain orders of magnitude more information than abstracts. From around 3,110 RegulonDB PubMed ids as of June 2006, we were able to obtain just 2,475 (79.6% of total). Even with those we actually collected, we also had to deal with incorrect conversion from PDF format, inconsistencies in term usage, etc. Another problem encountered is that not all the information is consistently presented in an explicit manner. Sometimes tables, graphics and illustrations provide what human curators need to generate relevant information, for example, by using some kind of domain inference in ways that our system was not designed to do. The sources used were also decisive factors in how well the system was able to generate a useful network. In order to compare the triage techniques employed in RegulonDB, we estimated what could be termed the "informational density" of the different corpora. We correlated the total size of the network obtained from these sources, the number of distinct documents and the raw size of each one, as shown in Table 3. This comparison allowed us to compare more accurately the quality and quantity of the network information obtained with each of the document sets. One of the ratios we estimated was the percentage of all interactions obtained that were found in RegulonDB (Column F), while other measures the average size of each document in the corpus (G), and the last one show how many RegulonDB interactions were obtained per document (H). The density of RegulonDB-related information shows that in a set of abstracts with fewer overall interactions than a full-text one with a similar number of documents, the relevant information can be more densely-packed, although we can expect to retrieve a smaller quantity of information. The interesting comparisons here are between documents gathered with different search strategies, and resulting in different numbers of documents. RegulonDB searches (RS) and STRING-IE abstract searches (ST) show a similar global network size, but quite different number of relevant interactions per document, while the curated EcoCyc paper set (EA) compares fairly well with RS. These numbers by themselves are not an explicit guideline of how to obtain a corpus that will maximize the potential information retrieved in Text Mining, but they do allow some degree of insight into diverse sources where document numbers and sizes as well as relevance are very different. Until the automatic paper selection issue is satisfactorily solved, high-throughput Information Extraction techniques can help lessen the impact of this specific problem on total results, since the technology can process equally well a lesser number of more informational papers than a much more extensive set of less-relevant papers, and still retrieve a significant amount of useful interactions.

**Table 3:** Informational density of various corpora

**A) Corpus**	**B) # Docs**	**C) Size (MB)**	**D) Interactions in RegulonDB**	**E) Total interactions**	**F) % In RegulonDB**	**G) Average doc size (kbs)**	**H) Interactions/Docs**
**RN**	724	24.9	1026	1643	62.4	65.9	1.41
**RP**	2475	99.0	1316	2650	49.6	40.0	0.53
**RA**	3075	3.3	322	414	77.7	1.07	0.1
**EA**	13334	14.4	402	627	64.1	1.08	0.03
**RS**	12059	12.3	400	718	55.7	1.02	0.03
**ST**	58312	10.7	342	691	49.5	0.18	0.005

A comparison of the degree of informativeness with regard to transcriptional regulation in *E. coli *K-12 in various corpora, as established from the number of RegulonDB-attested interactions they contain; The table includes total number of documents and interactions (Cols. B & E), percentage and number of all interactions found in RegulonDB (Cols. D & F), average size of each document in the corpus (Col. G), ordered by number of RegulonDB interactions per document (Col. H).

## Conclusion

How much valuable information is lost in papers that are filtered-out in the initial triage? Is a tightly-focused search-and-selection strategy better than casting a wider net? What is preferable when curating large-scale biological networks, exhaustiveness or precision in the data? Can automatic and manual annotation complement each other so we don't have to expect a trade-off with these two parameters? In this work we have addressed some of these issues by evaluating the use of NLP-based Information Extraction techniques for enhancing curation of a database of regulatory interactions. Saric *et al*. [15,16], for instance, presented a regulatory extraction system designed for inter-species versatility, but their evaluations focused more on the computational aspect of their system and not sufficiently on the implementation of such a system within a curatorial workflow. Rodriguez-Esteban *et al*. [20] evaluated the results of the GeneWays pathway Information Extraction system using Machine-Learning techniques to simulate the decision-making process of curators when reviewing the output of an IE system, thus framing the question as a classification problem ("correct/incorrect"). One conspicuous difference between their evaluation approach and ours is that we used RegulonDB as a gold standard, while they relied on manually-reviewed training-testing data, albeit with measures of inter-annotator agreement that ensures certain objectivity. Karamanis *et al*. [21] reported the use of NLP-based tools to assist curation of the fruit fly database, but their evaluations were based on the average time employed by curators to fill their forms and are thus not really comparable with our own methodologies for testing overall curation, where completion time is not as critical as annotation accuracy.

By going over automatically-curated information from an extensive spectrum of sources, human users can not only verify information already curated by more traditional means but can also encounter information that either escaped manual curation or can help contextualize previously captured information. An accurate pre-annotation of non-curated text is also possible with automatic processing, which can suggest, as well, better search heuristics for finding relevant literature. We believe that a parallel manual/automatic curation process, as shown in Figure 1, can achieve results that represent the best of both worlds. This two-pronged approach can ensure broad coverage of the data without significant loss of precision, since a human expert will still vet all information added to the reference database. At present we are evaluating the integration of NLP-aided tools to enrich and enhance RegulonDB curation. NLP tools will require a different type of curation, devoted initially to revising the false positives and negatives, in order to detect what failed in each case, thus providing feedback to the system developers in addition to the actual gathering of biological information, but not replacing manual curation.

Besides some of the challenges already mentioned in the previous section for a fully-automatic curation system, some of the system's shortcomings have to do with mundane reasons, such as incomplete named-entity dictionaries, imperfect format conversion, word tokenization, reporter-gene occurrences, etc. Incremental enhancement in these areas is time-consuming, but it is also perfectly feasible and does not represent especially difficult technical hurdles. A more complex challenge for automated curation is the interpretation of tables and figures, which help authors present multiple interactions or their evidence in a manner well suited for human readers, but enormously difficult for computer processing.

In this work we have put forward an xml-based markup language (RNM^2^L) to represent text-mined regulatory networks in a way that is useful both to biologists and to NLP specialists alike. We have also suggested using a combination of manual and automatic literature curation to achieve precision in the annotation without sacrificing completeness. Our experiences with automatic and semi-automatic annotation have shown that Natural Language Processing can be an extremely useful tool for curatorial efforts, although it is still far from being able to do full text interpretation or locate all the information that a trained human expert can. An important conclusion we have drawn is that since Information Extraction systems can as easily handle large amounts of documents as a more limited datasets, using extensive search strategies combined with more focused ones can provide both a satisfactory coverage of the potential information, as well as good precision, without too many false positives to review. A rule-based system will not obtain all the network interactions that a human curator team can, but will not overwhelm them with non-relevant instances, unlike a statistical-based one that can potentially be more comprehensive but also more noisy. Methods for filtering interactions that are based on, for example, the number of sentences attesting the same annotation or on the presence of known transcription factors, are being explored by our group and others [22], but the amount of interactions and the time needed to review them by curators in order to find overlooked or novel information, is still an interesting open question. Manual curation of the output of automatic processes is a good way to complement a more detailed review of the literature, either for validating the results of what has been already annotated, or for discovering facts and information that might have been overlooked at the triage or curation stages. By combining the exacting precision of human readers with the tireless abilities of Information Extraction systems to rapidly cover a lot of ground with reasonable accuracy, we believe that genomic data on other organisms less studied than *E. coli *can be obtained for the high-throughput methods of Systems Biology and Genomics.

## Methods

Each selected set of documents (a corpus) was first normalized and tokenized, separating all words and terms, and identifying individual sentences for processing. After this preprocessing stage, we tagged the Part-Of-Speech (POS) of each word using a customized version of Treetager [23], and then ran a retagging module that substituted some of the POS tags for more semantically-oriented labels, such as *org *(organism), *nnpg *(protein/gene name), *actv *(activation verb), etc., using both hand-crafted dictionaries and gene name lists created both by the RegulonDB team and by other groups, such as the original developers of the STRING-IE system. We also implemented a regular expression module for identifying genes and proteins that adhere to standard gene and protein naming conventions for *E. coli *(for instance, selecting a word that ended and started in upper case as a protein name candidate), and that used RegulonDB-specific identifiers for genes and proteins. Thus, in the phrase: "*nanC *expression is also activated by the regulators cyclic AMP-catabolite activator protein, OmpR, and CpxR", the system was able to identify OmpR and CpxR as proteins if the initial dictionary-based tagging missed them. Although we realize that an accurate terminological inventory and a robust ontology can go far in ensuring a correct interpretation of the textual content, we opted not to include in this version of the system a more sophisticated gene normalization algorithm based on Machine-Learning techniques, since pushing the envelope of Named-Entity Recognition was not as important as creating a reliable, baseline system with reasonable complexity as well as manageable processing resource usage. The resulting output combined syntactic and semantic tags, and was fed into a SCOL syntactic parser [24] that generated a tree-like structure by applying a biology-specific grammar, a finite-state formalism well known in Computational Linguistics. The resulting parse trees allowed for recognition of biological entities and processes in relationships inferred from the grammatical structure of the linguistic phrase, for example, relating two gene/protein noun phrases via a phrase containing verbs from a list of regulation-related lexemes, such as "up-regulates" or "binds". We modified the core grammars for the parser originally described in [15,16] in order to handle verbal phrase coordination and very simple anaphoric relationships involving some pronoun cases, which the original system didn't handled due to self-imposed restraints. Using these grammar extensions, three additional interactions corresponding to the repressing effects of ArcA, FruR and Fis on *acnB*, were extracted from previously mentioned sentence (1).

(1) In contrast, *acnB *expression is activated by CRP and repressed by ArcA, FruR and Fis from PacnB.

Chunk-parsed sentences were converted into an intermediate xml format and processed by customized heuristics modules that identify the regulatory interactions and establish, whenever possible, the kind of the interaction extracted (activation or repression). The complete system customized for *E. coli *can be downloaded from our server [19].

## Authors' contributions

CRP conceived the study, implemented the extraction system, carried out the automated evaluations and drafted most of the manuscript. HS provided and checked consistency of RegulonDB data and provided some of the figures. IMF carried out the curatorial review and performed biological interpretation of the results. JC-V motivated and coordinated the study and approved the final manuscript.
